# ﻿*Astermayangheense* (Asteraceae, *Aster*), a new species from Guizhou Province, China

**DOI:** 10.3897/phytokeys.257.145670

**Published:** 2025-06-10

**Authors:** Zhi Li, Jiang-hua Wu, Yu-jie Wang, Qi-xian Zhou, Ming-tai An

**Affiliations:** 1 College of Forestry, Guizhou University, Guiyang 550025, China Guizhou University Guiyang China; 2 Arts Institute, Guizhou Education University, Guiyang 550018, China Guizhou Education University Guiyang China; 3 Guizhou Mayanghe National Nature Reserve Administration, Yanhe County, Tongren City 565305, China Guizhou Mayanghe National Nature Reserve Administration Yanhe China

**Keywords:** *Aster*, morphology, molecular phylogeny, new taxon

## Abstract

*Astermayangheense* Z.Li (Asteraceae, Astereae), a new species from Guizhou, China, is described and illustrated here. Morphological and molecular analyses based on nuclear ribosomal DNA internal transcribed spacer (nrDNA ITS) confirm its distinct status. The species is morphologically similar to *A.saxicola*, but differs by its falcate, adaxially shiny upper leaves 3.5–5.0 × 0.8–2.0 cm with long-acuminate to caudate apices (versus oblong-lanceolate to lanceolate leaves in *A.saxicola*); 5-seriate, abaxially red-purple phyllaries (versus 3–5-seriate with purple-tipped phyllaries); and fewer florets (7–12 ray florets and 5–9 disc florets versus 9–14 ray florets and 10–18 disc florets). Phylogenetic analysis strongly supports its distinctness, with four samples forming a monophyletic clade (PP = 1.00, BS = 100%) nested within *Aster*.

## ﻿Introduction

The genus *Aster* L., comprises approximately 150 species, most of which are restricted to Eurasia (Ling et al. 1985; Chen et al. 2011; Li et al. 2012). To date, 123 species of *Aster* have been identified in China, with 75 of which are endemic (Chen et al. 2011; Li et al. 2012). South-western China, which includes the Qinghai–Tibet Plateau,Yunnan–Guizhou Plateau and Sichuan Province, harbours the highest species diversity of *Aster*, which makes this region a critical biodiversity hotspot for this genus (Li et al. 2012; Zhang et al. 2019). Many undescribed species have been found in this region in recent years (Li et al. 2017, 2020a, b; Xiao et al. 2019a, b; Xiong et al. 2019).

During our field investigation on the feeding habits of François’ Langur (*Trachypithecusfrancoisi*) in Mayanghe National Nature Reserve, Guizhou Province, China, in 2024, we found an unusual population of *Aster* growing on limestone canyons in the Mayang River Valley. Initially, the plant seemed similar to *Astersaxicola* W.P.Li & Z.Li in its basal and lower cauline leaves, procumbent or ascending stems and similar habitat. However, further careful examination revealed that several key diagnostic features of the plant differed from those of *A.saxicola*, such as its falcate, adaxially shiny upper leaves with long acuminate to caudate apices, its purple or red–purple, 5-seriate phyllaries and fewer florets. To determine the taxonomic status of this taxon, we conducted systematic molecular studies using nrDNA ITS sequence data. Both morphological and molecular evidence support that this distinct population represents a novel species; thus, we formally describe it here.

## ﻿Materials and methods

### ﻿Material collection and morphological observations

The morphological study was conducted, based on field-collected specimens and surveys in Mayanghe National Nature Reserve, located in Yanhe County, Tongren City, Guizhou Province, China. A total of 25 dried specimens and more than 10 living individuals of the newly-discovered species were examined for comprehensive morphological characterisation. Detailed observations and measurements were performed to document key diagnostic features. Voucher specimens were deposited in the Tree Herbarium of the Forestry College of Guizhou University (GZAC).

### ﻿Taxon sampling, DNA extraction, PCR reaction and sequencing

The sampled taxa, along with voucher details and GenBank accession numbers, are provided in Table 1, Appendix 1. Total genomic DNA was extracted from silica-gel-dried leaves using a modified CTAB method (Doyle and Doyle 1987). The nuclear ribosomal ITS region was amplified and sequenced using the primer pair ITS1 (5′-GTCCACTGAACCTTATCATTTAG-3′) and ITS4 (5′-TCCTCCGCTTATTGATATGC-3′) (Li et al. 2017, 2020a). PCR amplification were performed in a 25 µl volume containing 10 µl of template DNA, 12.5 µl of Taq PCR MasterMix (Sangon Biotech, China), 0.5 µl of each primer (10 µM) and 10.0–10.5 µl of sterile distilled water. The PCR amplification was performed following Li et al. (2020a): initial denaturation at 95 °C for 5 min; 30 cycles of 94 °C for 30 s, 56 °C for 30 s and 72 °C for 30 s; and a final extension at 72 °C for 8 min. PCR products were purified and sequenced by Tsingke Biotechnology Co., Ltd., Beijing, China.

**Table 1.** Morphological comparison of *Astermayangheense* and *A.saxicola.* Data for *A.saxicola* are sourced from Xiong et al. (2019).

Morphological Characters	*Astermayangheense*	*A.saxicola*
Rhizomes	5–20 cm long, short to long, sometimes slightly woody	3–10 cm long, short, slightly thickened, ± woody
Upper leaves	falcate, adaxially shiny, apex long acuminate to caudate; 3.5–5.0 × 0.8–2.0 cm	oblong-lanceolate to lanceolate, margin often entire, shortly petiolate to sessile; 1.0–6.7 × 0.4–1.5 cm
Phyllaries	5-seriate, abaxially red-purple or purple	3–5-seriate, purple or purplish at apex
Middle phyllaries (mm)	oblong or oblong-lanceolate, margin glabrous, 0.5–0.8 × 1.1–1.6	oblong, scarious margin ciliate, 2.1–3 × 1–1.2
Number of disc florets	5–9	10–18
Number of ray florets	7–12	9–14
Stigmatic appendages (mm)	long-triangular, 0.2–0.4 × 0.15–0.2	lanceolate, 0.6–1.2 × 0.2–0.35

### ﻿Phylogenetic analyses

NrDNA ITS sequences were used to explore the new taxon status in the *Aster* genus. The alignment of 53 sequences (Appendix 1: Table A1) from 50 species or varieties representing the major clade of *Aster* and its sister group. The aligned sequences ranged from 630 bp to 633 bp in length. *Dichrocephalaintegrifolia* (L.f.) Kuntze and *Grangeamaderaspatana* (L.) Poir. were designated as outgroups, following Li et al. (2012). Phylogenetic reconstruction was conducted using PhyloSuite_v.1.2.3 (Xiang et al. 2023). Maximum Likelihood (ML) analysis was performed with IQ-TREE v.1.6.12 (Nguyen et al. 2015) under the best-fit substitution model: General Time Reversible model with invariable sites and gamma distribution (GTR+I+G), selected via MrModelTest v.2.3 (Nylander 2004). Branch support was assessed using 1000 ultrafast bootstrap replicates. The resulting ML tree was manually adjusted in MEGA X (Kumar et al. 2018). Additionally, Bayesian Inference (BI) was implemented in MrBayes v.3.2.7 (Huelsenbeck et al. 2001), running two parallel Markov Chain Monte Carlo (MCMC) analyses for 10 million generations, sampling every 1000 generations. The first 25% of trees were discarded as burn-in and posterior probabilities were calculated from the remaining trees.

## ﻿Results

### ﻿Morphology study

The newly-described species, *Astermayangheense*, shares some morphological features with *A.saxicola*, such as broadly ovate to ovate basal and lower leaves (typically withered at anthesis), paniculate–corymbiform to corymbiform synflorescences and procumbent or ascending stems. Both species exhibit ecological specialisation, restricted to karst limestone crevices in canyon habitats. However, *A.mayangheensis* is unequivocally distinguished by its falcate, adaxially glossy upper leaves (3.5–5.0 × 0.8–2.0 cm) with long-acuminate to caudate apices (versus oblong-lanceolate to lanceolate leaves, 1.0–6.7 × 0.4–1.5 cm), 5-seriate red-purple to light purple phyllaries (versus 3–5-seriate phyllaries with purple/purplish only at apex), fewer florets for per capitulum (5–9 disc florets and 7–12 ray florets versus 10–18 disc florets and 9–14 ray florets, respectively) and a later flowering period (November–December versus September–October). These diagnostic characters are systematically compared in Table 1.

### ﻿Molecular phylogeny

Maximum Likelihood (ML) and Bayesian Inference (BI) analyses yielded similar tree topologies. The ML tree is presented in Fig. 4, with bootstrap support (BS) values and Bayesian posterior probabilities (PP) indicated at nodes. The four samples of *Astermayangheense* formed a strongly supported monophyletic clade (PP = 1.00, BS = 100%), which is nested within the redefined core *Aste*r clade of Eurasia (Li et al. 2012). Notably, the clade of *A.mayangheense* showed a weakly-supported sister relationship with *A.turbinatus* (BS = 62; PP < 0.50, omitted from the tree).

### ﻿Taxonomic treatment

#### ﻿ Aster mayangheense

Taxon classificationPlantaeAsteralesAsteraceae

Z.Li sp. nov.

039CDE0B-4A1E-5B82-8C3A-AF16FCFC4B82

urn:lsid:ipni.org:names:77363120-1

Figs 1 , 2

##### Diagnosis.

**Figure 1.**
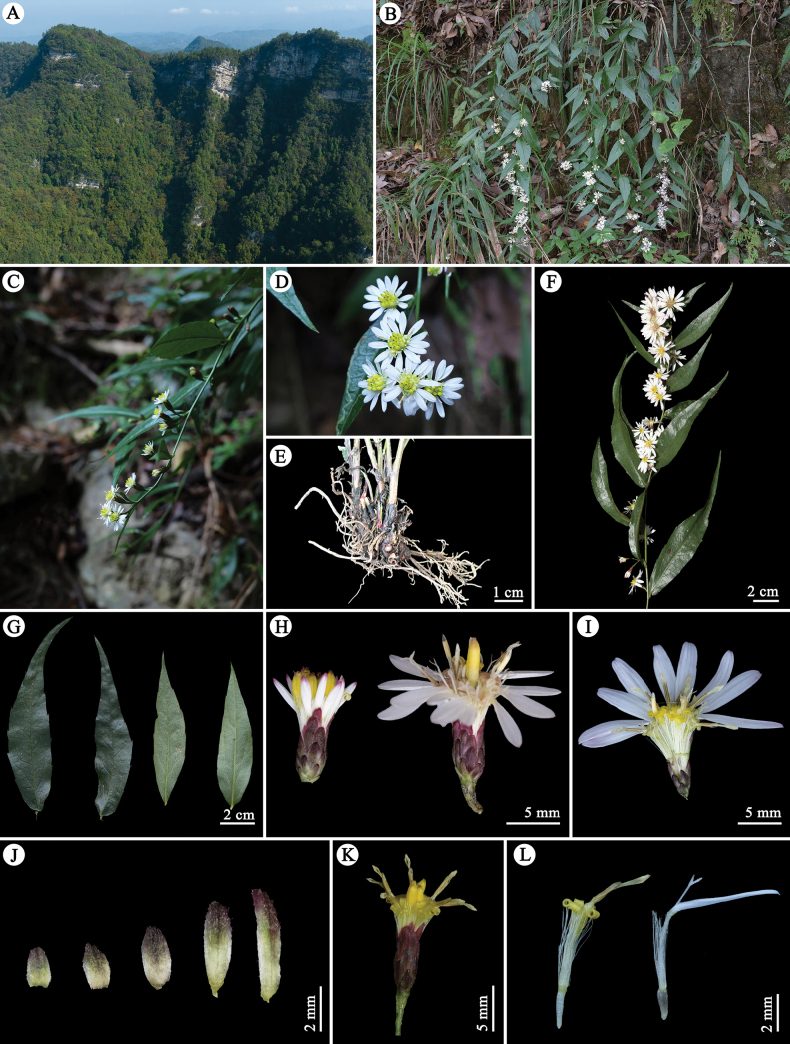
Habitat and morphology of *Astermayangheense***A** habitat **B** flowering plants **C** capitula arranged on a branch **D** top view of the capitula **E** rhizomes **F** upper leaves, falcate with shiny surface **G** side view of two capitula **H** phyllaries (from outer to inner, left to right) **I** a disc floret (left) and a ray floret (right).

**Figure 2.**
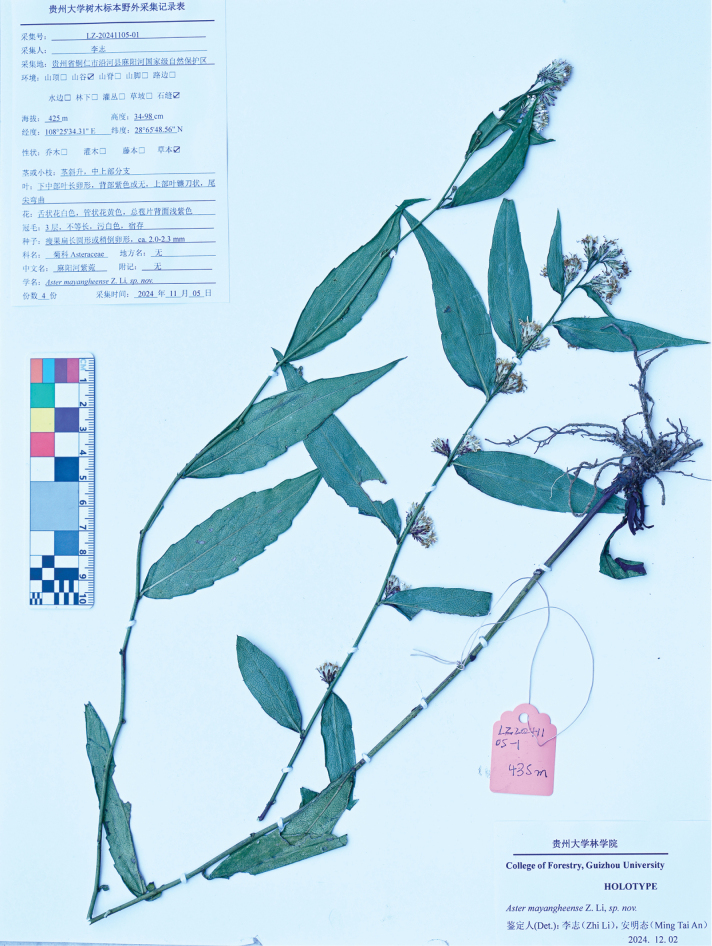
Holotype of *Astermayangheense*.

*Astermayangheense* differs from *A.saxicola* by its falcate upper leaves (3.5–5.8 × 0.8–2.0 cm) with adaxially shiny surfaces and long-acuminate to caudate apices (vs. oblong-lanceolate to lanceolate leaves 1.0–6.7 × 0.4–1.5 cm, margins entire, shortly petiolate to sessile), 5-seriate red-purple to purple phyllaries abaxially (vs. 3–5-seriate phyllaries purple/purplish only at apex) and fewer florets per capitulum (ray florets: 7–12 vs. 9–14; disc florets: 5–9 vs. 10–18) (Figs 1, 2, Table 1).

##### Type.

**Figure 3.**
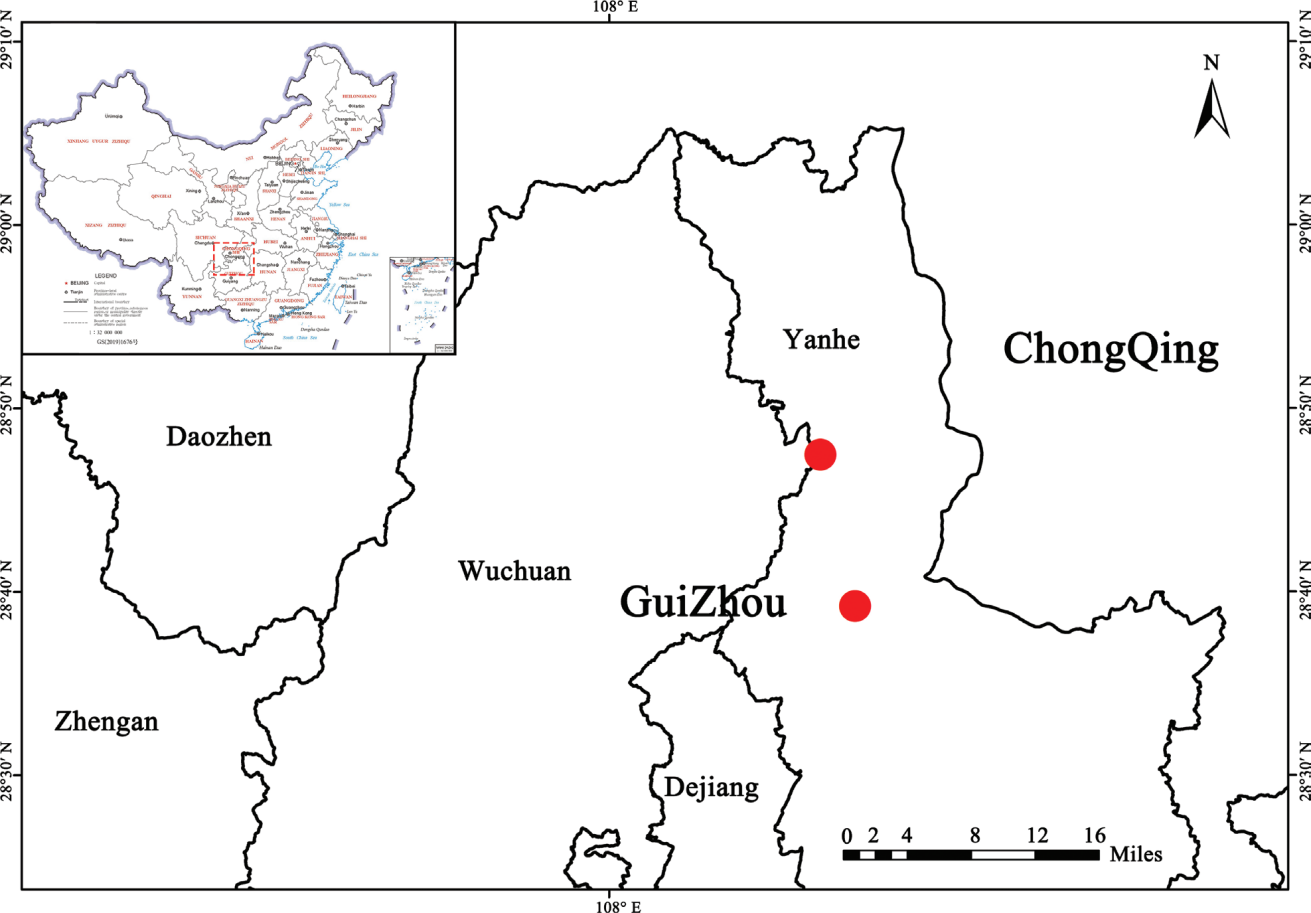
Distribution of *Astermayangheense* in the Mayanghe National Nature Reserve, Yanhe County, Guizhou Province, China (two red dots indicating localities).

China, Guizhou (贵州), Tongren City, Yanhe County, Mayanghe National Nature Reserve (Fig. 3), karst limestone canyon, alt. 435 m, 28.654856, 108.253431, 5 November 2024, *Zhi Li LZ20241105-1* (Holotype: GZAC!; isotypes: GZAC!) (Fig. 2).

##### Description.

Herbs, perennial, 30–100 cm tall. Rhizomes 5–20 cm long, sometimes somewhat woody. Stems caespitose, procumbent or ascending, some branching from the upper part or not, finely striated and glabrous above. Basal and lower cauline leaves withered at anthesis; basal leaves, broadly ovate or elliptic, 3.0–6.0 × 7.2–10.5 cm, abaxially puberulent, pale purplish or green, 3-veined, mid-vein prominent abaxially, margin sparsely serrate medially, petioles narrowly winged (1.0) 3.0–6.0 cm long. Middle cauline leaves sessile; blade papery, slightly thickened, oblong-oblanceolate to oblong-lanceolate, 4.2–15.0 × 2.8–4.8 cm, apex long-acuminate to caudate, adaxially shiny, sometimes abaxially purplish; mid-vein prominent abaxially; margin shallowly serrate with 3–4 (6) mucronate teeth per side. Upper leaves gradually reduced, falcate, 3.5–5.0 × 0.8–2.0 cm, adaxially shiny, abaxially green or faintly purplish, apex long-acuminate to caudate. Synflorescences terminal or axillary, paniculate-corymbiform to corymbiform, capitula;10–67 peduncles 5–10 mm. Involucres campanulate, 2.5–3.5 mm in diam.; phyllaries 5-seriate, imbricate, unequal, herbaceous, apex acute to acuminate, purple to red-purple; outermost phyllaries oblong, 0.5–0.7 × 1.1–1.5 mm; second series oblong to oblong-lanceolate, 0.5–0.8 × 1.1–1.6 mm; middle phyllaries oblong-lanceolate, 0.6–1.0 × 1.3–1.8 mm; fourth series narrowly oblong-lanceolate, 0.4–0.6 × 1.5–2.0 mm; innermost phyllaries narrowly oblanceolate, 0.5–1.0 × 2.0–2.5 mm. Ray florets 7–12, white; lamina elliptic, 4.1–9.2 × 0.5–1.2 mm, shallowly 3-lobed; tube 4.0–5.0 mm, glabrous. Disc florets 5–9, yellow; tube 2.0–3.1 mm, hairy, recurved; limb funnel-form; lobes lanceolate, ca. 1.6 mm, glabrous. Anthers triangular, ca. 0.8 mm. Achenes pale brown, oblong to slightly obovate, slightly compressed, 2.0–2.3 mm, strigose, 2-ribbed. Pappus 3-seriate, white to dirty whitish; outer bristles < 0.5 mm; middle bristles 2–6 mm; inner bristles ca. 4 mm. Flowering November–December.

**Figure 4.**
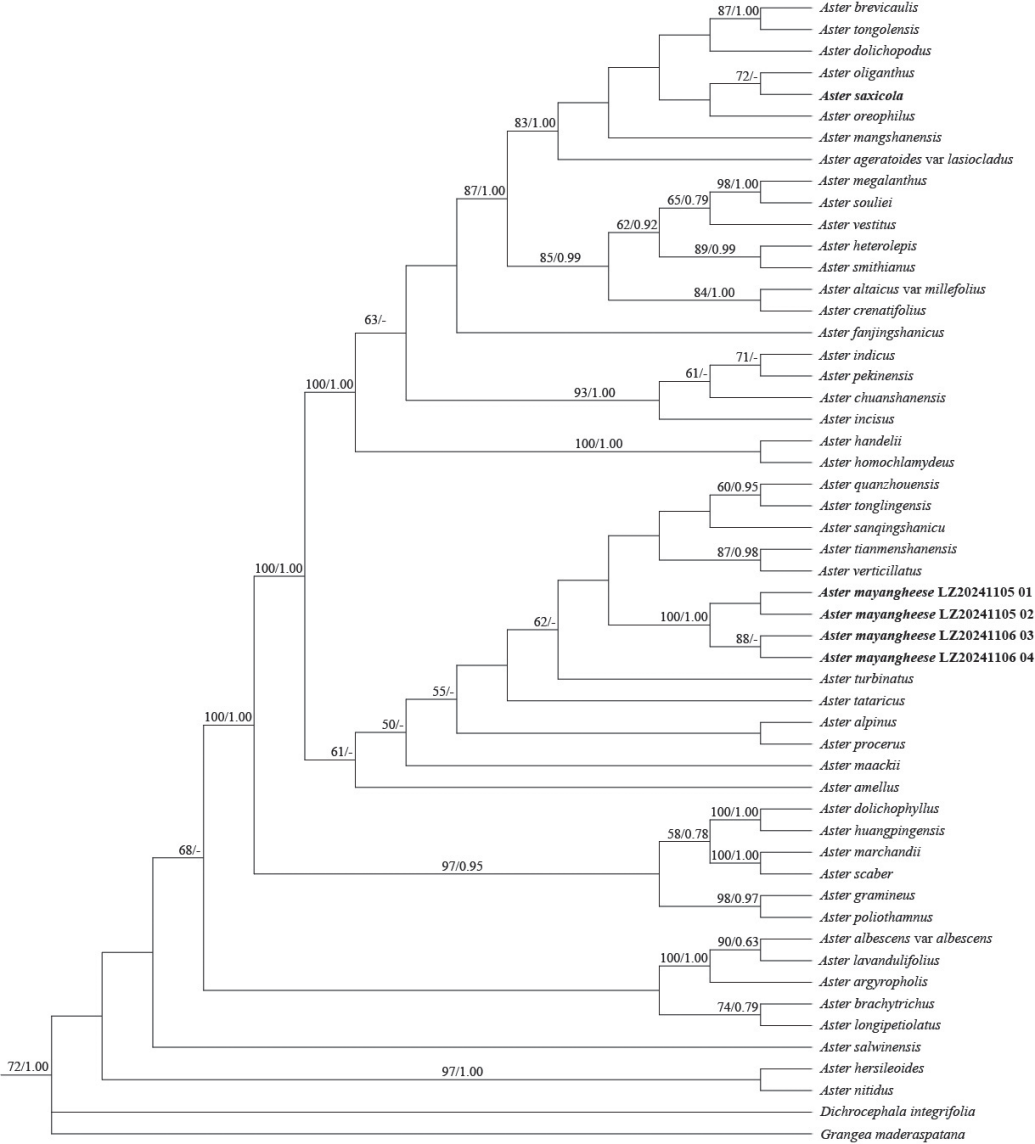
The Maximum Likelihood (ML) phylogram, based on ITS sequence data, showing the phylogenetic position of *Astermayangheense.* Nodal support values are indicated as ML bootstrap percentages (BS) and Bayesian posterior probabilities (PP); dashes (–) denote nodes with BS < 50% or PP < 0.5. The four samples of *A.mayangheense* and one sample of *A.saxicola* are highlighted in bold.

##### Etymology.

The specific epithet indicates the type locality, Mayanghe, Yanhe County, Guizhou Province, China. The locality name is rendered “Mayanghe National Nature Reserve” in Chinese Pinyin.

##### Distribution and habitat.

Endemic to karst limestone canyon (400–950 m a.s.l.) within Mayanghe National Nature Reserve, Yanhe County, Guizhou Province, China (Fig. 1A).

##### Phenology.

Flowering period November to December and fruiting period mid-November to December.

##### Vernacular name.

麻阳河紫菀 mā yáng hé zī wǎn in Chinese Pinyin.

##### Conservation status.

*Astermayangheense* is currently known from only one locality within the Mayanghe National Nature Reserve, a protected area characterised by karst limestone canyons with minimal anthropogenic disturbance. Due to the limited scope of field surveys, its conservation status has been assessed as Data Deficient (DD) under the IUCN Red List Categories and Criteria (IUCN 2022). However, this species plays a specific ecological role as a key food source for the wildlife conservation François’ Langur (*Trachypithecusfrancoisi*), suggesting that its persistence may face future threats if conservation measures are not prioritised. Further research is urgently needed to evaluate population trends, habitat requirements and potential risks.

##### Additional specimens examined (paratypes).

China • Guizhou, Tongren City, Yanhe County, Mayanghe National Nature Reserve, 05 November 2024, *LZ20241105-2*, *LZ20241105-3*, *LZ20241105-4* (deposited at GZAC).

## ﻿Discussion

The genus *Aster* represents a relatively young taxonomic group in Asteraceae currently undergoing rapid differentiation, characterised by complex morphological variation that renders it a classic “difficult group” in modern systematic studies. In this study, both morphological and molecular phylogenetic evidence unequivocally support the recognition of *A.mayangheensis* as a distinct species. Phylogenetic analyses, based on nrDNA ITS sequences, including 48 *Aster* species/varieties and two outgroup taxa from related genera, further resolved its systematic position within the core *Aster* clade. However, critical gaps remain in comprehensively delimiting its sectional placement in the genus *Aster*, particularly owing to the lack of cytological data (e.g. chromosome counts), chloroplast genomes and additional multilocus evidence. Future studies should integrate these datasets to precisely determine their taxonomic affinities within the genus.

Karst regions are widely recognised as “natural laboratories” for ecological and evolutionary studies, where their complex geomorphology forms habitat islands that support remarkable biodiversity and high levels of endemism. Guizhou Province, with karst landscapes covering more than 70% of its territory, is one of China’s most significant karst areas. Historically, limited transportation infrastructure has hindered comprehensive botanical surveys in remote regions. Recent improvements in accessibility have led to the discovery of numerous new endemic species within just the past five years, including *Asterhuangpingensis* (Li et al. 2020a), *Synotispanzhouensis* (Li et al. 2020b), *Ixeridiummalingheense* (Xu et al. 2024), *Salviapenghuana* (Qiu et al. 2024) and *Oxalisxishuiensis* (Yang et al. 2024), amongst others. These discoveries not only highlight the region’s remarkable species richness, but also strongly indicate the likely presence of numerous additional undescribed taxa in these unique karst ecosystems.

## Supplementary Material

XML Treatment for Aster mayangheense

## ﻿Additional information

### Author ORCIDs

Zhi Li https://orcid.org/0000-0001-7094-1672

Ming-tai An https://orcid.org/0000-0003-3886-0287

### Data availability

All of the data that support the findings of this study are available in the main text.

## ﻿Appendix 1

**Table A1.** Taxa sampled, vouchers and GenBank accessions.

Species name	Voucher information or reference	Accession number of ITS
A.albescensvar.albescens	Li et al. (2012)	JN543862
A.altaicusvar.millefolius	Li et al. (2012)	JN543709
A.ageratoidesvar.lasiocladus	Li et al. (2012)	JN543760
*Asteralpinus*	Li et al. (2012)	JN543817
*Asteramellus*	Li et al. (2012)	JN543817
*Asterargyropholis*	Li et al. (2012)	JN543793
*Asterbrevicaulis*	Xiao et al. (2019b)	MH638204
*Asterchuanshanensis*	Xiao et al. (2022)	MT731682
*Astercrenatifolius*	Li et al. (2012)	JN543823
*Asterdianchuanensis*	Xiao et al. (2019a)	MK693179
*Asterdolichophyllus*	Li et al. (2020a)	MH747068
*Asterdolichopodus*	Li et al. (2012)	JN543775
*Asterfalcifolius*	Li et al. (2012)	JN543844
*Asterfanjingshanicus*	Li et al. (2012)	JN543829
*Astergramineus*	Li et al. (2012)	JN315928
*Asterhandelii*	Li et al. (2012)	JN543820
*Asterhersileoides*	Li et al. (2012)	JN543787
*Asterheterolepis*	Li et al. (2012)	JN543823
*Asterhomochlamydeus*	Li et al. (2012)	JN543784
*Asterhuangpingensis*	Li et al. (2020a)	MH747070
*Asterincisus*	Li et al. (2012)	JN543715
*Asterlavandulifolius*	Li et al. (2012)	JN543796
*Asterlongipetiolatus*	Li et al. (2012)	JN315936
*Astermaackii*	Li et al. (2012)	JN543745
*Astermangshanensis*	Li et al. (2012)	JN543760
*Astermarchandii*	Xiao et al. (2019a)	MW419957
*Astermayangheense*	LZ20241105-01	PQ816786
*Astermayangheense*	LZ20241105-02	PQ816787
*Astermayangheense*	LZ20241106-03	PQ816788
*Astermayangheense*	LZ20241106-04	PQ816789
*Astermegalanthus*	Xiao et al. (2019b)	MK693187
*Asternitidus*	Li et al. (2012)	JN543790
*Asteroliganthus*	Li et al. (2017)	KY428860
*Asteroreophilus*	Li et al. (2012)	JN543826
*Asterpekinensis*	Li et al. (2012)	JN543718
*Asterpoliothamnus*	Li et al. (2012)	JN543763
*Asterprocerus*	Zhang et al. (2015)	KP313683
*Asterquanzhouensis*	Xiao et al. (2022)	ON055150
*Astersalwinensis*	Zhang et al. (2015)	KP313689
*Astersanqingshanica*	Xiao et al. (2022)	MW419955
*Astersaxicola*	Xiong et al. (2019)	MH936501
*Asterscaber*	Li et al. (2012)	JN315934
*Astersmithianus*	Li et al. (2012)	JN543778
*Astersouliei*	Li et al. (2012)	JN543826
*Astertataricus*	Li et al. (2012)	JN543748
*Astertianmenshanensis*	Zhang et al. (2015)	KP313677
*Astertonglingensis*	Zhang et al. (2019)	MH807119
*Astertongolensis*	Xiao et al. (2019a)	MK693184
*Asterturbinatus*	Li et al. (2012)	JN543814
*Asterverticillatus*	Li et al. (2012)	JN543706
*Astervestitus*	Li et al. (2012)	JN543769
*Dichrocephalaintegrifolia*	Li et al. (2012)	JN315919
*Grangeamaderaspatana*	Li et al. (2012)	JN315920
